# 

**Published:** 1919-03-08

**Authors:** 


### Medical Training and Infant Welfare.

In the House of Commons last week Mr. John Jones asked the President of the Board of Education whether, in view of the supreme national importance of rearing a generation of healthy children, he will take steps to make instruction in the medical conditions of childhood a regular part of medical training in universities and medical schools. The answer given by Mr. Fisher was that instruction in the medical and surgical diseases of children forms an integral part of the medical curriculum in all medical schools receiving the Board's grants.

### His Majesty and Hospital Saturday Fund.

Lord Stamfordham has written from Buckingham Palace to the promoters of the London Hospital Saturday Fund as follows: "His Majesty, as patron of the fund, is gratified to hear of the eminently satisfactory result of last year's collection, securing as it did the largest amount collected by the association since its inception. The King heartily congratulates the officials and honorary workers upon their achievement, which is especially praiseworthy when the difficulties due to war conditions under which their efforts have been carried out are realised." The fund received £70,913 in 1918, over £12,000 more than in 1917.

### The College of Nursing (Limited by Guarantee).

DERBY CENTRE.

(Hon. Secretary, Miss HAINES, Children's Hospital.)

A GENERAL MEETING will be held at the Derbyshire Royal Infirmary, Derby, on Thursday, March 13, at 3 P.M. All members are cordially invited.

LIVERPOOL CENTRE.

(Hon. Secretary: Miss C. WORSLEY, Matron, Infirmary for Children.)

THE next meeting will be held in the lecture theatre of the Liverpool University (entrance through the Royal Infirmary) on Wednesday, March 12, at 6.15 P.M. The lecturer will be Major Thurstan Holland, L.R.C.P., M.R.C.S., and the subject "X-ray and Orthopædics." The lecture will be demonstrated by slides. It is earnestly requested that every member will attend, as the meeting will be followed by a discussion on the coming election of the College of Nursing.

NEW CENTRE IN SUSSEX.

AT a well-attended meeting at the Royal Sussex County Hospital, Brighton, a resolution was passed that a Brighton and Hove Centre be established; the Executive Committee was appointed, and a date fixed for the first meeting and the appointment of officers. Sir Thomas Butler, K.C.V.O., presided, and was supported by Miss K. Scott (the matron) and Mr. R. Ball Dodson (chairman of the hospital). Miss Cowlin (Organising Secretary of the College) said the College of Nursing was not a trade union, for the simple reason that no self-respecting nurse could use the weapon of a trade union by laying down tools and refusing to nurse the sick, but it could give weight to the nurse's claim for just treatment, and was already making this weight felt in many directions.

### SEAMEN'S HOSPITAL SOCIETY.

Owing to the expected large attendance, the annual meeting on Friday, March 7, will be held at His Majesty's Theatre, at 3 p.m., instead of at Princes Restaurant as previously announced.

### Matrons' and Sisters' Appointments.

Addenbrooke's Hospital, Cambridge.—Miss I. C. Wilkinson has been appointed night sister. She was trained at the Royal Hants County Hospital, Winchester, later being theatre sister there, and has since been sister at the Essex County Hospital, Colchester, and at the Norfolk and Norwich Hospital, Norwich.

Ear and Throat Hospital, Birmingham.—Miss Pauline Fitzner has been appointed theatre sister. She was trained at the Ashton-under-Lyne District Infirmary and Children's Hospital; has been staff nurse at the Children's Infirmary, Liverpool; and sister at the Auxiliary Hospital, Myrtle Street, Liverpool.

County Hospital, Dorchester.—Miss Alice G. Atkins has been appointed sister. She was trained at the Gloucester Royal Infirmary and Eye Institution; has been night and out-patients' sister at the Hospital for Nervous Diseases, Welbeck Street, London; and ward sister at the Royal Victoria and West Hants Hospital, Boscombe, and at the Taunton and Somerset Hospital.

General Hospital, Nottingham.—Miss Dorothy Ibbotson has been appointed *x*-ray sister. She was trained at the Queen's Hospital, Birmingham, and has been out-patient sister at the Warneford Hospital, Leamington.

City of Westminster Union Infirmary, Hendon.—Miss A. J. Edwards has been appointed sister. She was trained at Medway Union Infirmary, where she has since been ward sister.

Hospital for Women, Liverpool.—Miss Margaret Wynn Jones has been appointed theatre sister. She was trained at King Edward VII.'s Hospital, Cardiff, where she was later staff and theatre nurse, and has been sister at Chester War Hospital.

Carnarvonshire and Anglesey Infirmary, Bangor.—Miss Elizabeth Hughes has been appointed sister. She was trained at the Infirmary and Dispensary, Warrington, and at the Monsall Fever Hospital, has been staff nurse and temporary sister at the former institution, and staff nurse at the Welsh Metropolitan War Hospital, Whitchurch, Cardiff.

General Hospital, Maesteg.—Miss Frances M. Hollister has been appointed matron. She was trained at King Edward VII.'s Hospital, Cardiff, has been matron of the Tredegar Park Hospital, Tredegar, the Miners' and Women's Hospital, Redruth, and of the St. Leonard's Hospital, Sudbury, Suffolk. Miss Hollister has also done military nursing in Queen Alexandra's Imperial Military Nursing Service Reserve.

Maesteg and District Cottage Hospital.—Miss E. Hore has been appointed sister. She was trained at the Wirral Infirmary, Bebington, Birmingham, has been sister at the Military Hospital, Torquay, and has served under the British Red Cross Society.

West Cornwall Miners' and Women's Hospital, Redruth.—Miss Mary Lugg has been appointed sister. She was trained at the above institution, where she has since been staff nurse.

Bradwell Isolation Hospital, Chesterton.—Miss Elizabeth C. Rugg has been appointed matron. She was trained at the Royal Infirmary, Huddersfield, and for three and a half years held the position of matron at the Borough Isolation Hospital, Stafford.

General Hospital, Burton-on-Trent.—Miss Elsie Dickens has been appointed ward sister. She was trained at Warnford, Leamington, and South Warwickshire Hospital, where she afterwards engaged in private nursing. Subsequently she was sister at the Children's Hospital, Sheffield. Miss Edith Cullen has been appointed night sister. She was trained at the Royal Victoria Hospital, Dover, where she was afterwards sister.

### Gifts and Bequests.

Mr. Walter Browne, who is a member of Exeter City Council, has given £1,000 to the Royal Devon and Exeter Hospital.

Princess Alice, Countess, of Athlone, has received £300 through the Dowager Countess Brassey, and £50 each from Lord Newlands and Mr. Edward Yate for the fund she is raising to provide new accommodation for the nurses of Middlesex Hospital.

The Clothworkers' Company has sent its annual subscription of fifty guineas as well as an extra donation of fifty guineas to the Royal Hospital and Home for Incurables, Putney.

Mr. Joseph Prior, Senior Fellow of Trinity College, has bequeathed £100 to Addenbrooke's Hospital, Cambridge.

Sir Charles Ernest Tritton, of Norwood, S.E., bequeathed £150 to Norwood Cottage Hospital and £100 to the British Home and Hospital for Incurables.

Cardiff Infirmary benefits to the extent of £20,000 under the will of Viscount Rhondda.

A cheque for £3,400 has been sent to the British Red Cross Society by the Queen of the Belgians as the proceeds of the concert given recently in the Albert Hall by the Belgian Field Orchestra.

Lord Ashton, of Lancaster, has sent to the secretary of the Preston and County of Lancaster Queen Victoria Royal Infirmary a cheque for £2,000 to wipe out the deficit on the maintenance account. He also sent a handsome annual subscription.

Mrs. Gertrude Augusta Johnson, of Earl's Court, formerly of Wytham-on-the-Hill, Lincs, bequeathed £1,000 to St. George's Hospital, London.

Sir Ernest Cassel has placed in the hands of trustees for Educational purposes, the sum of £500,000.

Mr. John Rankin, shipowner, has given £20,000 to the Liverpool Cathedral Building Fund.

Bequests of £200 to St. George's Hospital, London, and £100 each to Hanwell Cottage Hospital and Worthing Hospital were made by Miss Mary Elizabeth Rennell, of Egerton Place, S.W.

### Personalia.

Sir Nestor Tirard has been appointed consulting physician to King's College Hospital, London, and has been elected Emeritus Professor of Medicine by the council of the College.

Capt. W. I. Cumberlidge, R.A.M.C.(T.)M.B., F.R.C.S., senior assistant hon. surgeon, has been appointed honorary surgeon to the Leicester Royal Infirmary. He has recently returned from service in France, but is not yet demobilised.

Mr. Huw R. Gruffydd has been appointed acting assistant secretary to the Great Northern Central Hospital in succession to Mr. Joseph Sharpe, who has taken up duties as manciple to Charterhouse.

Dr. Henry John Strong, of Worthing, whose death is announced at the age of eight-six, was consulting surgeon to the Croydon General Hospital, where he was formerly surgeon, and honorary consulting physician to the Freemason's Institution at Croydon.

